# Using Residual Blood from the Arterial Blood Gas Test to Perform Therapeutic Drug Monitoring of Vancomycin: An Example of Good Clinical Practice Moving towards a Sustainable Intensive Care Unit

**DOI:** 10.1155/2022/9107591

**Published:** 2022-12-27

**Authors:** T. J. L. Smeets, D. van de Velde, B. C. P. Koch, H. Endeman, N. G. M. Hunfeld

**Affiliations:** ^1^Erasmus MC University Medical Center Rotterdam, Department of Hospital Pharmacy, Doctor Molewaterplein 40, 3015 GD Rotterdam, Netherlands; ^2^Erasmus MC University Medical Center Rotterdam, Department of Intensive Care, Doctor Molewaterplein 40, 3015 GD Rotterdam, Netherlands

## Abstract

**Background:**

Regarding sustainability in the intensive care unit (ICU), there is increasing interest in reducing material waste and avoiding unnecessary procedures. Therapeutic drug monitoring (TDM) of vancomycin, using a dedicated tube, is standard clinical care during treatment with vancomycin. Furthermore, in the ICU, on a daily basis, arterial blood gas (ABG) tests are frequently performed throughout the day. After analysis, a variable volume of blood is discarded. Lithium heparin (LiHep) syringes for ABG tests differ from normally used dipotassium ethylenediaminetetraacetic acid (K_2_EDTA) tubes. The primary objective was to compare both containers and validate the use of LiHep syringes. Secondary objectives were to evaluate the potential impact on saving materials, nursing time, and costs when implementing vancomycin TDM via LiHep syringes.

**Methods:**

Vancomycin analysis from sampling in lithium heparin (LiHep) syringes for ABG tests was validated and compared with the concentrations from conventional sampling in K_2_EDTA tubes. For method comparison, a Bland–Altman plot and Deming regression analysis were performed. The method was validated for inter- and intra-day precision and accuracy. Vancomycin was analyzed by means of the validated method using a particle-enhanced turbidimetric inhibition immunoassay (PETINIA) autoanalyzer. Furthermore, an analysis was conducted to evaluate the potential impact of implementing vancomycin sampling via ABG tests on savings in materials, nursing time, and costs.

**Results:**

From 18 patients, 24 plasma samples in both K_2_EDTA tubes and LiHep syringes were obtained and compared. The mean relative difference between the two containers was −2.0% (−3.0 to −0.93%). Both the Deming regression analysis and the Bland–Altman plot met the acceptance criteria. Potentially, over 1000 blood draws and accompanying materials and packaging can be saved when vancomycin samples are obtained by means of scavenged LiHep syringes. The vancomycin analysis for LiHep syringes showed a total interday precision of 1.95% and an accuracy of 99.7%. The total intraday precision was 2.22%, and the accuracy was 99.2%. Accuracy and precision values were within the acceptance criteria of recovery 85 to 115% and ≤15%, respectively.

**Conclusion:**

No significant differences were found in vancomycin concentration between the two analyses, and the LiHep analysis was validated for further implementation in clinical care. Residual blood from ABG test samples can be used for TDM of vancomycin, resulting in a potential reduction of materials used and the number of blood draws. These results will contribute to a more sustainable TDM process with benefits for the patient.

## 1. Introduction

Within hospitals, the intensive care unit (ICU) generates a considerable amount of waste due to the large amount of materials used [1–3]. This is mainly due to the patient's complexity and their needs, which require extensive equipment, procedures, and materials. In addition, many clinical procedures are routinely performed due to habit and could therefore contribute to unnecessary waste production [4–7]. However, the aim to reduce waste production must be balanced against the need to maintain optimal patient care.

A common and repeating procedure is therapeutic drug monitoring (TDM) [8]. The traditional sample collection for TDM is conventionally performed by venipuncture or directly from the indwelling arterial or central venous line and requires an extra blood draw and several accompanying materials. In order to pursue a more sustainable TDM process and reduce the use of materials, scavenged sampling seems to be an attractive strategy.

Scavenged sampling involves the use of residual materials of all biological fluids that are leftover from standard clinical care practice. It does not carry any extra burden or risk for the patient. Moreover, this strategy has the additional benefit to minimize required patient blood volumes. Notably, scavenged sampling does not require additional materials for the sampling process. In a recent review, Schouwenburg et al. have shown that scavenged sampling is a promising sustainable sampling strategy to introduce a sustainable mindset in clinical practice and research [9].

Arterial blood gas (ABG) tests are another illustrative example of routine clinical procedures and, at the same time, a potential method to apply scavenged sampling [10]. ABG tests are frequently performed throughout the day in order to monitor the patient's vital parameters. After analysis, a syringe with a variable volume of blood is discarded. Similarly, the adjustment of vancomycin dosing based on TDM is a standard clinical procedure during treatment with vancomycin [11]. Vancomycin is frequently used in critically ill patients for the treatment of life-threatening infections, including methicillin-resistant *Staphylococcus aureus* [11]. The specifics of the dipotassium ethylenediaminetetraacetic acid (K_2_EDTA) tube for vancomycin sampling in our clinical setting differ from those of lithium heparin (LiHep)-coated syringes for ABG tests.

In this study, as the first elaboration of a possible example for the clinical use of scavenged sampling in the ICU, we aim to compare both containers, validate the use of LiHep syringes, and determine whether scavenging residual blood from ABG tests is suitable for a more sustainable TDM process of vancomycin in the ICU.

## 2. Methods

### 2.1. Method Comparison

Regular vancomycin samples from ICU patients receiving continuous vancomycin infusion were collected in K_2_EDTA tubes obtained from standard clinical care for TDM. Simultaneously, leftovers from LiHep syringes used for standard clinical care blood gas analysis were scavenged for vancomycin analysis. By default, LiHep syringes for ABG analysis are labeled by the ICU nurse with a patient identification label. Subsequently, in this study, the nurse recorded the time of sampling on the label. Afterwards, the labeled syringe was directly handed over to the dedicated researcher for vancomycin analysis in the hospital's pharmacy laboratory. Vancomycin concentrations in K_2_EDTA tubes and LiHep syringes were compared based on the Clinical and Laboratory Standards Institute (CLSI) EP09 guideline for method comparison studies [12]. The medical research ethics committee approved the study and waived informed consent. A Bland–Altman plot and Deming regression analysis for method comparison were performed by using GraphPad Prism version 9.0.0 (GraphPad Software, San Diego, CA, USA). For the Bland–Altman plot, all samples should be within a 10% error. For Deming regression, if the 95% confidence interval (CI) for the intercept contains the value 0, it is concluded that there is no constant difference between the two containers. When the 95% CI for the slope includes the value 1, it is concluded that there is no proportional difference between the two containers.

### 2.2. Validation

Importantly, for further implementation of scavenged LiHep samples into clinical practice, it is recommended to validate this new method. Since only containers are different between the two samples, a partial validation of LiHep syringes was performed according to the CLSI EP05 guidelines [13]. Precision and accuracy were estimated with 4 measurements per day over a 5-day period for 3 different vancomycin concentrations. In every analysis, 3 levels of quality control (QC) (12.2, 30.8, and 64.3 mg/L) were analyzed. QC 12.2 mg/L and QC 30.8 mg/L were realized from scavenged LiHep patient samples. QC 64.3 mg/L was prepared by adding a vancomycin stock solution in deionized water to a scavenged LiHep patient sample. According to the CLSI guidelines, the vancomycin analysis for LiHep syringes was considered acceptable if the intraassay and interassay with LiHep syringes did not exceed 15% recovery with precision within ±15% (relative standard deviation, RSD). No matrix effect analysis was performed; since in both containers, the matrix serum and sample preparation remain equivalent.

### 2.3. Chemicals and Materials

Vancomycin HCl was purchased from Cayman Chemicals (Ann Arbor, MI, USA). Deionized water was prepared using a Milli-Q® Advantage A10® purification system from Merck Millipore (Darmstadt, Germany). Drug-free human serum was obtained from the Hemostasis Laboratory of Erasmus MC, University Medical Centre (Rotterdam, Netherlands). Becton Dickinson K_2_EDTA tubes and LiHep syringes were used for blood collection (Franklin Lakes, NJ, USA).

### 2.4. Vancomycin Analysis

Before analysis, the researcher verified that simultaneous sampling collection was obtained within 4 hours and that blood was not hemolysed. When the blood was hemolysed or 4 hours between the collection and analysis was exceeded, the samples were discarded and not used for analysis. Blood from LiHep syringes was transferred into a tube for further analysis at the hospital's pharmacy laboratory. All blood samples were collected and centrifuged at 1811*g* for 6 min. Vancomycin was analyzed by the validated method using a particle-enhanced turbidimetric inhibition immunoassay (PETINIA) autoanalyzer, Abbott Architect C4000 (Chicago, IL, USA) and Abbott vancomycin assay (6E44-21, Chicago, IL, USA) were used. The TDM Abbott multiconstituent calibrator was used to calibrate the vancomycin assay (05P0401, Chicago, IL, USA).

### 2.5. Cost, Material, and Nurse Time Analyses

The costs of the necessary materials for TDM of vancomycin were obtained from the hospital financial support office. To evaluate an estimate of total costs, we summed the costs and multiplied them by the number of vancomycin samples in 2021. Furthermore, nurses were asked to indicate the time spent on traditional vancomycin sampling. Nurse labor costs were calculated by multiplying the estimated labor time by the average hourly wage.

## 3. Results

From 18 patients, 24 plasma samples in both K_2_EDTA tubes and LiHep syringes were obtained and compared. The K_2_EDTA samples ranged from 12.4 to 29.8 mg/L, and the corresponding LiHep samples ranged from 13.1 to 30.5 mg/L. The mean relative difference was −2.0% (−3.0 to −0.93%).

**Figure 1:**
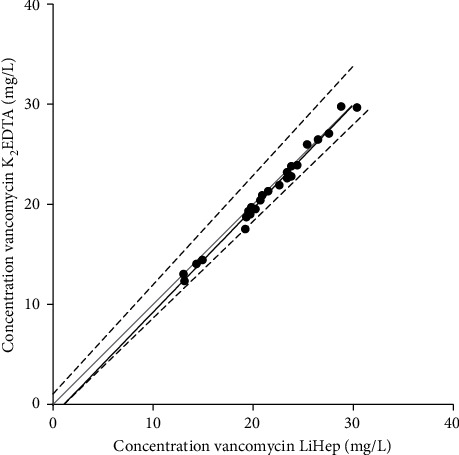
Deming regression between vancomycin concentrations for conventional K_2_EDTA tubes and LiHep syringes. The black line, black-dashed lines, and grey line represent the Deming regression, the 95% confidence interval, and the unity line *Y* = *X*, respectively. LiHep, lithium heparin; K_2_EDTA, dipotassium ethylenediaminetetraacetic acid; mg/L, milligram per liter.

**Figure 2:**
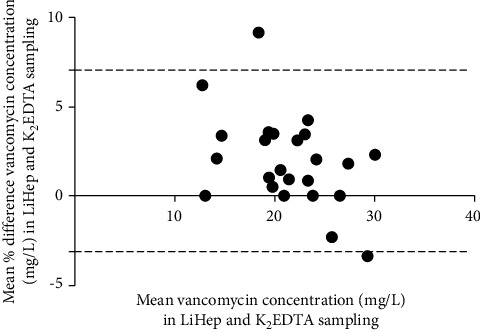
Bland–Altman plot of the differences in vancomycin concentrations from LiHep and K_2_EDTA samples. The black-dashed lines represent the 95% confidence interval. LiHep, lithium heparin; K_2_EDTA, dipotassium ethylenediaminetetraacetic acid; mg/L, milligram per liter.

Figure 1 shows the Deming regression analysis. The regression equation was *Y* = 0.9662*X* + 1.089, where *Y* represents the vancomycin concentration for LiHep syringes and *X* represents the vancomycin concentration for K_2_EDTA tubes. The CI for the intercept and the slope was −1.132 to 0.9482 and 0.9702 to 1.100, respectively. Both the slope and intercept met the acceptance criteria.  Figure 2 shows the agreement between vancomycin samples for LiHep syringes and K_2_EDTA tubes with the 95% confidence interval. All samples were within a 10% relative error and suggested good agreement between the two different containers.

Table 1 shows the validation results of interday and intraday accuracies and precisions for three levels of QC samples. Accuracy and precision values were within the acceptance criteria of recovery 85 to 115% and ≤15%, respectively.

Traditional vancomycin sample collection from an indwelling central line requires a K_2_EDTA tube, vacutainer connector, needle, and laboratory label. The material cost for one vancomycin sample is approximately €0.50. In 2021, a total of 214 patients in our ICU were treated with vancomycin. The number of TDM samples was 1072 with a median of 3 samples per patient. The result shows that when using scavenged LiHep syringes, a reduction in accompanying materials and packaging with a value of about €536 is reported. Our nurses indicate that every traditional sample collection, from collecting materials to sending the sample, takes approximately 10 minutes of nursing time. The average hourly wage for ICU nurses is €57. With 1072 vancomycin samples in 2021, it is possible to economize €10184 in nurse labor costs on the annual basis.

## 4. Discussion

In this study, in critically ill patients, a method comparison was performed between scavenged vancomycin samples in LiHep ABG syringes and conventional K_2_EDTA tubes. No clinically relevant differences were found in vancomycin concentrations between two different containers. The result shows that residual blood obtained from LiHep syringes for ABG tests is suitable for TDM of vancomycin. Subsequently, in order to legitimately apply the quantification of vancomycin, a partial validation of the scavenged LiHep samples was executed. All validation parameters were within the preset specifications. With these results, we performed the first two steps of the proposed workflow by Schouwenburg et al. (application, validation, and realization) to implement scavenged sampling for TDM in clinical practice.

To our knowledge, this is the first study that described scavenged sampling of vancomycin. Schouwenburg et al. performed a literature search on scavenged drug sampling but did not find any studies on scavenged sampling of vancomycin. The authors found several bridging studies, which compared the statistical performance of scavenged sampling versus scheduled sampling for population pharmacokinetic analysis. None of those studies fully elaborated on the total process of scavenged sampling for TDM. Ideally, method comparison and validation are performed before the scavenged sampling method is applied to research or clinical practice. In this study, we tried to give full transparency and detailed information about a possible approach to implementing scavenged sampling for TDM of vancomycin into clinical practice in the ICU. Certainly, the underlying thought behind this study is to contribute to a more sustainable ICU without compromising the quality of patient care. This study is the first act of “start small, think big” and may serve as an example of sustainable clinical care practice.

We found an estimated reduction in a cost of approximately €536. Nonetheless, potentially all vancomycin samples could have been obtained by using scavenged LiHep syringes from ABG tests, saving 1000 tubes, connectors, needles, labels, and packaging materials.

Another important ethical aspect and benefit to the patient is the reduction of potentially 1000 ICU phlebotomy moments by using scavenged sampling. Currently, clinical practice involves collecting high volumes of blood per patient several times a day. In our study, we observed a variable blood waste of 0.3 to 3.0 mL per ABG test, which is more than sufficient to analyze vancomycin. Bodley et al. presented a mean bedside waste per blood draw of 3.9 mL from the arterial lines and 5.5 mL from the central venous lines [14]. However, repetitive and high-volume phlebotomy is not without any risk. Bodley et al. also showed that a higher average daily phlebotomy volume is associated with ICU-acquired anemia [14]. In addition, every additional intervention on the arterial or central venous line involves the risk of introducing line infection. Therefore, pairing TDM and diagnostic sampling could reduce the phlebotomy number and volume. Whitehead et al., who showed that bundled interventions could reduce the volume of blood loss among adult ICU patients by approximately 70%, confirmed our assumption [15].

Furthermore, every traditional sample collection takes approximately 10 minutes of the nursing time and could annually save over €10000 in labor costs. With this in mind and the global nurse shortage, a reduction in unnecessary procedures may contribute to a more sustainable commitment to our nurses. Therefore, when you collect and combine TDM samples for other anti-infective drugs via scavenged sampling, an even more significant reduction in waste, nursing time, and accompanying costs is expected without compromising patient care.

As a next step, in order to pursue a more sustainable TDM process and reduce the use of materials, we intend to implement scavenged sampling into clinical practice. Additionally, as the following step, the application of scavenged sampling via ABG tests to other drugs for TDM can be studied as well. For further implementation, it is worth noting that the administrative and infrastructural procedures due to scavenged sampling will differ from conventional sampling for TDM. Only the vancomycin drug was studied, whose stability in whole blood and plasma has already been demonstrated [16, 17]. Therefore, no additional analyses were performed to study the stability of vancomycin. Therefore, to implement scavenged sampling for other drugs, it is important to take preanalytical stability and handling procedures into consideration, but for vancomycin, stability was not an issue. Additionally, in this study, there was a dedicated researcher responsible for the infrastructural procedures and ICU nurses had to record the time of collection for the LiHep syringe. A next step would be to explore the possibility of safely using a pneumatic tube system to directly transport the LiHep syringes to the hospital pharmacy laboratory. Furthermore, in order to prevent preanalytical errors, the labeling of LiHep syringes should be automated by using the electronic healthcare information system.

Some limitations of the study should be noted. Only samples of continuous vancomycin infusion with concentrations of 12.4 to 30.2 mg/L were compared and validated. Since no clinically relevant differences were found in vancomycin concentration in both containers and the vancomycin assay was already validated for linearity in K_2_EDTA tubes, no relevant deviation was expected outside these concentrations. In our opinion, continuous administration of vancomycin facilitates the use of scavenged sampling because compared to intermittent infusion, the sampling time is not dependent on the following dose. Moreover, the CLSI guideline recommends 40 samples for method comparison. However, the required number depends on the objective of the type of method comparison. Vancomycin was already validated for K_2_EDTA tubes, and therefore, only containers are different between the two samples. No outliers were found between the two samples, so no additional sampling was collected.

## 5. Conclusion

Altogether this study shows that residual blood from ABG test syringes can be used for TDM of vancomycin, the result shows a reduction in the materials used and also demonstrates that no extra blood draw is needed. These results may serve as an example for TDM of other drugs in order to realize a sustainable TDM process in the ICU in combination with benefits for critically ill patients.

## Figures and Tables

**Table 1:** Interday and intraday accuracy and precision of QC samples of vancomycin from LiHep samples.

Sample	Interday		Intraday	
	Accuracy (recovery, %)	Precision (RSD, %)	Accuracy (recovery, %)	Precision (RSD, %)
QC 12.2 mg/L	100.4	1.8	97.9	2.5
QC 30.8 mg/L	100.4	1.7	100.0	1.5
QC 64.3 mg/L	98.3	2.4	99.8	2.6

QC, quality control; LiHep, lithium heparin; RSD, relative standard deviation.

## Data Availability

Data are available from the corresponding author upon request.
